# Efficiency trends of essential public health services and possible influencing factors since the new round health reform in China: a case study from Hainan Province

**DOI:** 10.3389/fpubh.2023.1269473

**Published:** 2023-11-06

**Authors:** Ye Tian, Jia Peng, Yumei Liu, Jiayan Huang

**Affiliations:** ^1^International School of Public Health and One Health, Hainan Medical University, Haikou, China; ^2^Key Lab of Health Technology Assessment, National Health Commission, School of Public Health, Fudan University, Shanghai, China

**Keywords:** public health service, efficiency, data envelopment analysis, Malmquist productivity index, Tobit regression

## Abstract

**Objective:**

This article aimed to evaluate the efficiency trends and influencing factors of essential public health services in Hainan Province after the healthcare reform launched in 2009 in China.

**Methods:**

The efficiency of essential public health services (EPHS) at primary health institutions was assessed using data envelopment analysis (DEA), and the efficiency change was analyzed by employing the Malmquist productivity index (MPI). We used Tobit regression to identify the influence of environmental factors on the efficiency of public health services. The bootstrap method was adopted to reduce the impact of random errors on the result.

**Results:**

The bootstrapping bias-corrected efficiency revealed that the average values of technical efficiency, pure technical efficiency, and scale efficiency were 0.7582, 0.8439, and 0.8997, respectively, which meant that the EPHS in Hainan Province were not at the most effective state. The average bias-corrected MPI was 1.0407 between 2010 and 2011 and 1.7404 between 2011 and 2012. MPIs were less than 1.0000 during other periods investigated, ranging from 0.8948 to 0.9714, indicating that the efficiency of EPHS has been decreasing since 2013. The Tobit regression showed that the regression coefficients of *per capita* GDP, population density, the proportion of older people aged over 65, and the proportion of ethnic minority population were 0.0286, −0.0003, −0.0316, and − 0.0041 respectively, which were statistically significant (*p* < 0.05).

**Conclusion:**

There was a short-term improvement in the efficiency of EPHS in Hainan after the launch of the new round of health reform. However, this trend has not been sustained after 2013. In particular, equalized financial investment in essential public health could not fulfill the needs of poor counties. This has resulted in the inability to improve scale efficiency in some counties, which in turn has affected the improvement of overall EPHS efficiency. Therefore, to promote EPHS efficiency sustainably, it is suggested that under this model of provincial control of counties, the equity of resource allocation should be effectively improved while further advancing the technology of service delivery.

## Introduction

Non-communicable diseases (NCDs) have posed threats to the health of the population, especially children, pregnant women, and older people. NCDs are responsible for 70% of global mortality and 67% of deaths in lower- and middle-income countries (1). Primary health care (PHC), which provides basic health services for all people, is significant in meeting the challenge of NCDs. Hence, WHO updated the Declaration of Alma-Ata in 2018 and identified PHC as the driving force for achieving the Sustainable Development Goals (2). Countries around the world have responded to the declaration. For instance, some countries are undergoing reforms in the health system to solve the problem that the health system is overly hospital-centric and public health services are in short supply at the grassroots level (3, 4). Similarly, efforts have been made by several countries since the Declaration of Alma-Ata to expand the coverage of public health services, reduce health inequalities, and tackle the growing burden of NCDs (5–7).

China has paid great attention to PHC as well. With economic development and population aging, China is facing a change in the disease spectrum. The main diseases that affect mortality in China have shifted from infectious diseases to NCDs during the last 3 decades. The Chinese government has also emphasized the role of PHC in the prevention and control of NCDs and provided strong policy support in areas including service delivery, health financing, and governance (8). To meet the growing demand for public health services, a new round of healthcare reform, which established a government-led diversified investment mechanism, was launched in 2009. The reform specifically emphasized “ensuring the basics and strengthening the grassroots” and increased investment in primary healthcare institutions. Funded by the central and local governments, primary healthcare institutions provide a package of free services for residents, including immunization, health education, NCDs management, maternal and child health management, etc., which are known as essential public health services (EPHS). The number of primary healthcare institutions in China increased from 882 thousand in 2009 to 978 thousand in 2021, with a compound annual growth rate of 0.86%. In addition, the government raised the minimum standard of *per capita* fiscal subsidies for EPHS from US$ 2.2 to US$ 12.25 in the same time period. However, more research is needed to know whether these investments have improved the efficiency of EPHS.

Stochastic frontier analysis (SFA) and Data envelopment analysis (DEA) are commonly used in research to measure efficiency. The SFA is a parameter method that consider random errors, but it can only include one output variable in the model. On the other hand, the DEA is a non-parameter method and it is applicable for efficiency evaluation with multiple input and output variables. Since the health system provides multiple health services, the DEA would be more suitable for this study. For example, a study conducted in the Nordic countries combined constant returns to scale DEA and variable returns to scale DEA to evaluate the efficiency of university hospitals. Subsequently, ordinary least squares were used to analyze the influence factors of the efficiency (9). However, other scholars assumed that Tobit regression is more suitable as efficiency scores based on the DEA are censored data: scholars in the United States utilized an input-oriented DEA model and a cross-sectional time-series Tobit regression to assess the association between hospital ownership and technical efficiency (10). A DEA model assuming constant returns to scale was employed to analyze health systems in 29 OECD countries and panel Tobit analysis was proposed to obtain consistent and unbiased estimators in the second stage (11). In addition, a study carried out in Greece evaluated the efficiency of public hospitals by using Bootstrapping DEA and truncated regression procedures (12). These studies evaluating the efficiency of health systems were mainly conducted in developed countries, and studies conducted in developing countries were relatively few (13).

With regard to China, the Slacks-based Measure DEA model and Tobit regression were used to calculate the efficiency of public hospitals in Guangdong Province and identify key influence factors (14). Studies in Hunan Province applied input-oriented BCC DEA and the Tobit model to estimate the efficiency of healthcare institutions and their influence factors (15). Scholars employed the DEA model to evaluate the efficiency of public and private hospitals in Beijing and used the panel Tobit regression model with random effect to analyze the influence factors of the efficiency (16). As commonly used statistical models, DEA and Tobit regression can evaluate the efficiency and its influencing factors in a relatively accurate way. However, this model cannot measure the change in efficiency over time and ignores the influence of random error.

Therefore, bootstrap and Malmquist productivity index were added in this study, based on the traditional two-stage DEA model (DEA and Tobit regression). Considering that the existing research mainly focuses on the efficiency evaluation in the medical field of economically developed countries or regions with relatively little focus on the efficiency of public health services (13, 17), the objective of this study is to evaluate the efficiency of EPHS and its influence factors after China launched the new health reform in 2009. Feasible policy implications will be drafted to help further decision-making. This study hopes to provide references for future studies in other countries or regions.

## Materials and methods

### Sample site

There are 34 provincial-level administrative regions in China. Hainan Province is located in the south of China, with a land area of 35,400 square kilometers and a resident population of 10.3 million. The management of NCDs in Hainan Province has the following two characteristics. Firstly, according to the related data of key NCDs, the adjusted prevalence of total diabetes in Hainan Province was 17.5% (18). The healthy life years free from NCDs in Hainan Province was 77.60 years in 2015, which was close to the national data of 77.25 years in China (19). Thus, the management of NCDs in Hainan Province approximates the national average level.

Secondly, Hainan Province implements a system of provincial management of counties. There are a total of 3 cities and 15 counties in the province, including six autonomous counties for ethnic minorities. There are four levels of the administrative region in China: province, city, county, and township. However, the counties in Hainan are directly affiliated to the province, instead of the city, in terms of finance and administration due to small geographic areas and population size. This kind of management has saved administrative costs and enhanced the provincial government’s ability for overall planning (20). Therefore, health resources can be more efficiently distributed to primary healthcare institutions, which undertake significant responsibilities in the prevention and control of NCDs due to the provincial management of counties.

### Variables and data sources

It is essential to select a suitable group of input and output variables in order to accurately measure the efficiency of EPHS. Input variables for this study were chosen in terms of finance and labor. The first input was the financial allocation for essential public health, which served as a proxy for financial investment, and the second input was the number of health technicians representing labor investment. According to the national guidelines of EPHS, the following 5 services provided each year were selected as output variables in this study: the number of health records for residents, the number of 0-6-year-old children under the National Immunization Program (NIP), the number of health records for women in pregnancy at the first trimester, the number of patients under standardized management of hypertension, and the number of patients under standardized management of diabetes.

EPHS were also influenced by other social determinants. This study considered the following environmental variables: *per capita* GDP, urbanization rate, population density, and the proportion of people aged over 65. There are 56 ethnic groups in China, and besides the Han ethnic group, the other 55 ethnic groups are all minorities. Since ethnic minorities account for 17.8% (2022) of the population in Hainan Province, the proportion of the ethnic minority population was selected in this study, too.

The input and output variables were collected from administrative data for the total of 330 community health service centers and township hospitals in Hainan Province from 2010 to 2021. All required data on environmental factors were obtained from the Hainan Statistical Yearbook.

### Statistical analysis

The DEA model was adopted in this study. This model is a linear, non-parametric approach used to compare the efficiency among decision-making units (DMUs). The relative efficiency of DMUs can be obtained by constructing the efficiency frontier, which represents the highest level of efficiency of DMUs within a period. There are two important assumptions in the efficiency measurement of DEA.

The first one is the scale assumption. In 1978, the first model was proposed by Charnes, Cooper, and Rhodes (CCR model). This model is based on constant returns to scale (CRS), which means that the output will change in the same proportion as the input (21). The outcome of CRS is referred to as technical efficiency (TE). Later on, Banker, Charness, and Cooper extended the CCR model by adopting the assumption of variable returns to scale (VRS) (22). This model was named as BCC model and it measures the efficiency under different returns to scale. Based on CRS, the outcome of VRS separates TE into pure technical efficiency (PTE) and scale efficiency (SE), as shown in the following equation:


TE=PTE×SE

SE reflects the gap between the current size and the optimal size. The values of TE, PTE, and SE range from 0 to 1. The DMU is more efficient as the efficiency values approach 1. Considering that the amount of public health services produced per unit of input may change as inputs increase, the BCC model was adopted in this study to obtain comprehensive results.

The choice of orientation is another important assumption when performing DEA. Input-oriented models focus on the extent to which each input should be reduced to achieve efficiency with constant output; Output-oriented models focus on the extent to which each output should increase in order to be effective with constant input. In this study, an input-oriented model was employed because (1) health resource inputs are more manageable than outputs; (2) the health sector is more interested in how to reduce costs instead of maximizing the output since the amount of EPHS depends on the essential public health demands of the population (23).

Since DEA can only measure the relative efficiency in a period, the Malmquist productivity index (MPI) was adopted to provide an approach to measure the change of efficiency over time. There are several ways to decompose MPI, according to the different variants of the formula. This study chose the classical method proposed by Fare et al. in 1994 (24). Fare et al. divided MPI into two parts: technical efficiency change (TEC) and technical change (TC). The technical efficiency change can be further decomposed into pure technical efficiency change (PTEC) and scale efficiency change (SEC), as shown in the following equation:


$$\begin{array}{c} M_{0}\left(x_{t+1},y_{t+1},x_{t},y_{t}\right)=\sqrt{\frac{D_{0}^{t}\left(x_{t+1},y_{t+1}\right)}{D_{0}^{t}\left(x_{t},y_{t}\right)}\times \frac{D_{0}^{t+1}\left(x_{t+1},y_{t+1}\right)}{D_{0}^{t+1}\left(x_{t},y_{t}\right)}}\\ =\frac{D_{0}^{t+1}\left(x_{t+1},y_{t+1}\right)}{D_{0}^{t}\left(x_{t},y_{t}\right)}\times \sqrt{\frac{D_{0}^{t}\left(x_{t+1},y_{t+1}\right)}{D_{0}^{t+1}\left(x_{t+1},y_{t+1}\right)}\times \frac{D_{0}^{t}\left(x_{t},y_{t}\right)}{D_{0}^{t+1}\left(x_{t},y_{t}\right)}}\\ =TC\left(x_{t+1},y_{t+1},x_{t},y_{t}\right)\times TEC\left(x_{t+1},y_{t+1},x_{t},y_{t}\right)\\ \# \end{array}$$

where x and y are the inputs and outputs in t or t + 1 period; $\frac{D_{0}^{t}\left(x^{t+1},y^{t+1}\right)}{D_{0}^{t}\left(x^{t},y^{t}\right)}$ and $\frac{D_{0}^{t+1}\left(x^{t+1},y^{t+1}\right)}{D_{0}^{t+1}\left(x^{t},y^{t}\right)}$ uses distance function to, respectively, measure technology at period *t* and *t + 1*.

TEC, PTEC, and SEC represent the direction and degree of changes in technical efficiency, pure technical efficiency, and scale efficiency, respectively. TC reflects the change in the efficiency frontier. If the change is greater than 1, it indicates an increase in efficiency. If the change is equal to or less than 1, it means efficiency is unchanged or has decreased.

Moreover, the traditional DEA model ignores random errors and environmental factors, which may cause bias in efficiency values and change. Thus the bootstrap method was used to obtain bias-corrected results by repeating the sample 2000 times.

Since the efficiency value calculated by the DEA model ranges from 0 to 1, we employed the maximum likelihood estimation of the Tobit regression model to assess the impact of environmental factors on the efficiency of EPHS. The mixed Tobit regression model and random effects Tobit regression model for panel data are typical Tobit regression models. The model that fits the data better can be ascertained by the likelihood ratio test (LR test) (25). The Tobit model was as follows:


$$\theta =\beta_{0}+\beta_{1}X_{1}+\beta_{2}X_{2}+\beta_{3}X_{3}+\beta_{4}X_{4}+\beta_{5}X_{5}+\varepsilon$$

where θ is bootstrapping bias-corrected TE, $X_{1\sim 5}$ are five environmental variables mentioned above, and ε is the error term. The significance level was set to 0.05. Variance inflation factors (VIFs) were calculated to test the multi-collinearity problem.

In this study, all 18 cities/counties in Hainan Province were designated as the DMUs. MaxDEA8.0 software was used for DEA and stata16.0 was used for Tobit regression analysis.

ArcGIS Desktop 10.8 was employed to create figures in results.

## Results

### Overview of inputs, outputs, and environmental factors on EPHS

There are two input variables. The mean financial allocation for public health per county/city increased from US$ 0.75 million in 2010 to US$ 4.78 million in 2021. The mean number of health technicians per county/city increased from 371 in 2010 to 714 in 2021.

On the output side, the mean number of health records for residents per county/city increased by an average of 17.06 thousand per year, from 162.54 thousand in 2010 to 350.16 thousand in 2021. The mean number of patients under standardized management of hypertension per county/city increased by an average of 1.85 thousand per year, from 2.22 thousand in 2010 to 22.61 thousand in 2021. The mean number of patients under standardized management of diabetes per county/city increased by an average of 0.81 thousand per year, from 0.42 thousand in 2010 to 9.38 thousand in 2021. The mean number of 0-6-year-old children under the National Immunization Programme (NIP) per county/city increased annually from 2010 to 2012 and decreased in the following 8 years. The mean number of health records for women in pregnancy at the first trimester per county/city increased from 3.29 thousand in 2010 to 4.87 thousand in 2013 and subsequently decreased to 3.62 thousand in 2021 (Table 1).

**Table 1:** The means of input and output variables for 18 DMUs during 2010–2021.

	Inputs		Outputs				
	The number of health technicians	Financial allocation for public health (million)	The number of health records for residents (thousand)	The number of 0-6-year-old children under the national immunization program (NIP) (thousand)	The number of health records for women in pregnancy at the first trimester (thousand)	The number of patients under standardized management of hypertension (thousand)	The number of patients under standardized management of diabetes (thousand)
2010	371	0.75	162.54	97.81	3.29	2.22	0.42
2011	391	1.20	192.01	102.38	3.74	4.00	0.74
2012	433	1.50	259.51	129.52	4.77	13.59	4.28
2013	473	1.63	290.53	122.34	4.87	16.48	5.34
2014	489	1.71	295.17	118.92	4.67	17.16	5.99
2015	504	2.10	299.67	116.49	4.38	17.27	6.33
2016	526	2.11	306.88	107.75	4.42	16.51	6.33
2017	571	2.53	290.98	105.92	4.30	18.24	6.85
2018	567	3.07	299.20	104.79	3.91	17.11	6.50
2019	605	2.71	306.98	97.09	3.79	16.89	6.62
2020	649	3.78	324.79	90.03	3.93	17.33	6.85
2021	714	4.78	350.16	88.94	3.62	22.61	9.38

As to the environmental factors in Hainan Province, the annual average *per capita* GDP per county/city was US$ 5.53 thousand. The highest annual average *per capita* GDP was US$ 9.40 thousand in Sanya, and the lowest was US$ 3.51 thousand in Wuzhishan. The annual average urbanization rate ranged from 33.62% in Ledong to 77.70% in Haikou. The annual average population density ranged from 66.74 people/km^2^ in Qiongzhong to 1080.30 people/km^2^ in Haikou. The annual average proportion of people aged over 65 ranged from 5.43% in Sanya to 13.98% in Wenchang. The annual average proportion of the ethnic minority population ranged from 0.18% in Lingao to 71.60% in Wuzhishan (Appendix Table 1).

### Efficiency values of EPHS

The average values of technical efficiency, pure technical efficiency, and scale efficiency in Hainan Province between 2010 and 2021 were 0.8334, 0.9109, and 0.9160, respectively. After adjusted by the bootstrapping bias-corrected efficiency, the average values changed to 0.7582, 0.8439, and 0.8997, respectively (Table 2). There was a decrease in the efficiency values after correction, indicating that they were overestimated due to the interference of random errors.

**Table 2:** Original efficiency and bootstrapping bias-corrected efficiency of each county/city.

	Original efficiency			Bias-corrected efficiency		
	TE	PTE	SE	TE	PTE	SE
Haikou	0.6440	0.9788	0.6574	0.6019	0.8807	0.6814
Sanya	0.8868	0.9244	0.9601	0.8254	0.8632	0.9567
Danzhou	0.8910	1.0000	0.8910	0.8248	0.9009	0.9172
Wuzhishan	0.8618	0.9932	0.8645	0.7638	0.8969	0.8506
Qionghai	0.9140	0.9376	0.9730	0.8348	0.8688	0.9597
Wenchang	0.7247	0.9117	0.8097	0.6805	0.8548	0.8131
Wanning	0.9722	1.0000	0.9722	0.8448	0.8987	0.9410
Dongfang	0.9136	0.9915	0.9210	0.8310	0.9215	0.9015
Ding’an	0.8702	0.9287	0.9380	0.8188	0.8869	0.9247
Tunchang	0.9893	0.9962	0.9930	0.8768	0.9128	0.9605
Chengmai	0.9737	0.9754	0.9981	0.8737	0.9018	0.9687
Lingao	0.9771	1.0000	0.9771	0.8616	0.9043	0.9538
Baisha	0.7107	0.8357	0.8553	0.6671	0.8033	0.8363
Changjiang	0.7629	0.7961	0.9550	0.6965	0.7568	0.9212
Ledong	0.7370	0.7799	0.9508	0.6879	0.7355	0.9381
Lingshui	0.6370	0.6674	0.9542	0.5984	0.6451	0.9269
Baoting	0.8748	0.9606	0.9023	0.7465	0.8679	0.8543
Qiongzhong	0.6605	0.7193	0.9144	0.6129	0.6898	0.8890
Mean	0.8334	0.9109	0.9160	0.7582	0.8439	0.8997

**Figure 1:**
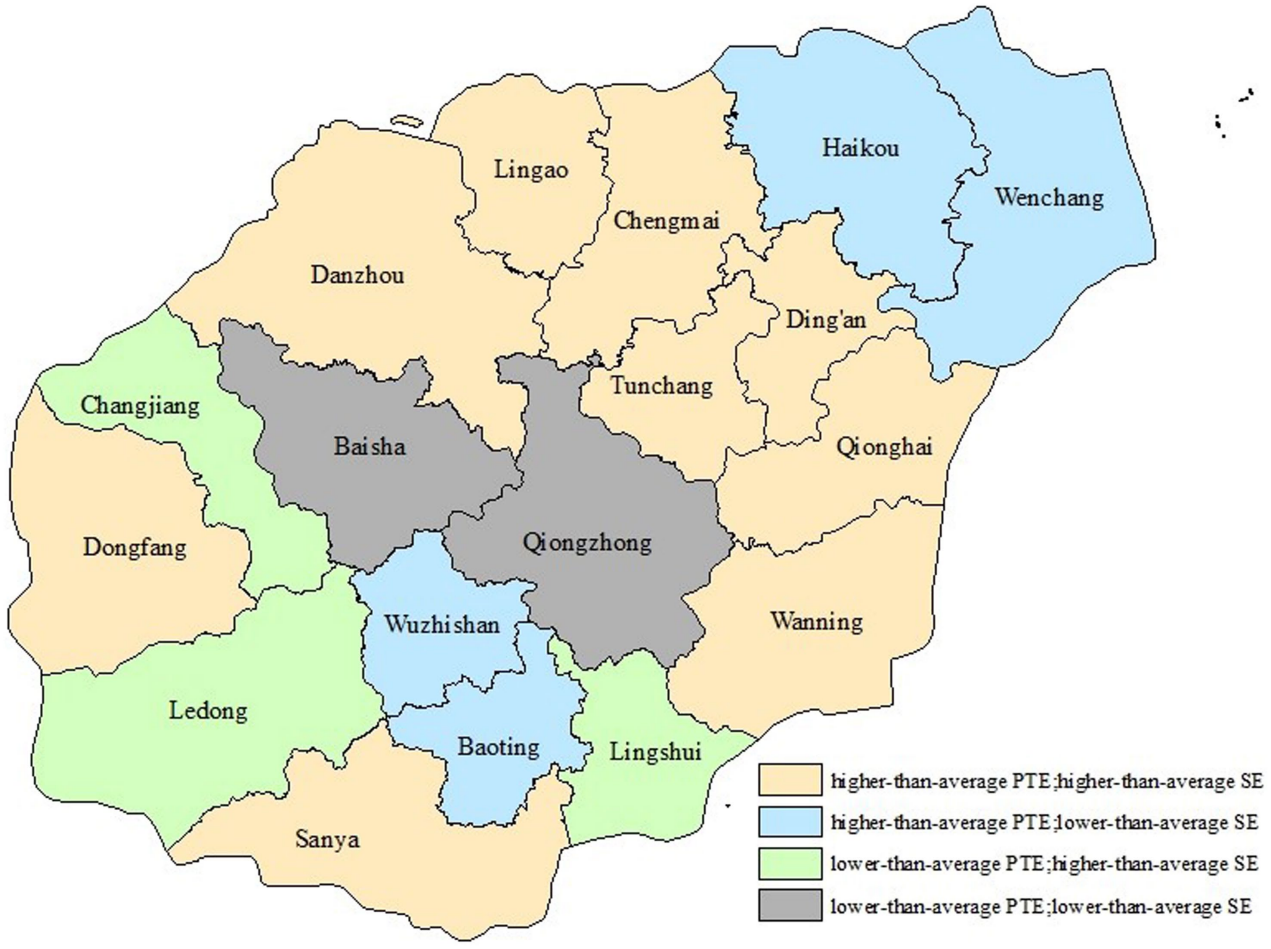
Four groups classified based on bootstrapping bias-corrected PTE&SE.

Although the average values of pure technical efficiency and scale efficiency were similar, there was apparent spatial variation in pure technical efficiency and scale efficiency, and the 18 DMUs can be separated into four groups based on the bootstrapping bias-corrected pure technical efficiency and scale efficiency. The first group included Sanya, Danzhou, Qionghai, Wanning, Dongfang, Ding’an, Tunchang, Chengmai, and Lingao, all of which had higher-than-average pure technical efficiency and scale efficiency. The efficiency values of DMUs in this group were generally higher than those of other groups. The second group included Changjiang, Ledong, and Lingshui, which had lower-than-average pure technical efficiency and higher-than-average scale efficiency. This shows that the efficiency of DUMs in this group was limited by the former. The third group embraced Haikou, Wuzhishan, Wenchang, and Baoting which had higher-than-average pure technical efficiency and lower-than-average scale efficiency. This indicates that the efficiency of DMUs in this group was restrained by scale efficiency. Lastly, Baisha and Qiongzhong were in the fourth group, with both efficiencies lower than the average (Figure 1).

### Changes of EPHS efficiency

The 18 DMUs’ average bias-corrected MPI, technical efficiency change, technical change, pure technical efficiency change, and scale efficiency change during 2010–2021 were 1.0003, 1.0061, 0.9942, 1.0029, and 1.0031, respectively. These suggested that the efficiency of EPHS in Hainan Province had increased slightly from 2010 to 2021 although there was a decrease in technical change. Notably, an improvement in MPI was observed between 2010 and 2011, with an improvement of 16.73% in technical efficiency change and a regression of 10.84% in technical change. Another advancement in MPI was observed between 2011 and 2012, with an improvement of 80.50% in a technical change and a regression of 3.58% in technical efficiency change. Except for 2010–2012, the MPIs were less than 1.0000 during other periods investigated, the value of which ranged from 0.8948 to 0.9714, indicating that the efficiency of EPHS has been decreasing since 2013 (Table 3).

**Table 3:** Bootstrapping bias-corrected MPI and its components during 2010–2021.

	MPI	TEC	TC	PTEC	SEC
2010–2011	1.0407	1.1673	0.8916	1.1640	1.0029
2011–2012	1.7404	0.9642	1.8050	0.8674	1.1117
2012–2013	0.9554	1.1559	0.8265	1.1437	1.0107
2013–2014	0.9414	0.9417	0.9996	0.9334	1.0089
2014–2015	0.8948	0.9059	0.9878	0.9217	0.9829
2015–2016	0.9497	1.0162	0.9345	1.0077	1.0085
2016–2017	0.9284	0.9657	0.9614	0.9474	1.0194
2017–2018	0.9714	1.0468	0.9280	1.0724	0.9761
2018–2019	0.9424	0.9091	1.0366	0.9599	0.9471
2019–2020	0.9493	0.9987	0.9505	1.0348	0.9652
2020–2021	0.8980	1.0323	0.8699	1.0222	1.0098
Mean	1.0003	1.0061	0.9942	1.0029	1.0031

According to the regional difference of changes in the 18 DUMs, 8 DMUs had an MPI less than 1.0000, which was mainly caused by the regression in technical change. The other 10 DMUs had an improvement in MPI from 2010 to 2021 (Table 4).

**Table 4:** Bootstrapping bias-corrected MPI and its components of 18 counties/cities.

DMU	MPI	TEC	TC	PTEC	SEC
Haikou	0.9826	1.0353	0.9491	1.0013	1.0339
Sanya	0.9887	1.0336	0.9565	1.0304	1.0031
Danzhou	0.9892	1.0017	0.9876	1.0054	0.9963
Wuzhishan	1.0055	0.9474	1.0613	1.0011	0.9463
Qionghai	1.0802	1.0360	1.0427	1.0256	1.0101
Wenchang	1.0944	1.0731	1.0198	1.0013	1.0717
Wanning	1.0560	1.0113	1.0442	1.0011	1.0102
Dongfang	1.0107	1.0134	0.9974	1.0006	1.0128
Ding’an	1.0314	0.9947	1.0369	1.0086	0.9862
Tunchang	0.9211	1.0051	0.9164	1.0008	1.0043
Chengmai	1.0224	0.9976	1.0249	1.0025	0.9951
Lingao	1.0091	1.0096	0.9995	1.0035	1.0061
Baisha	1.1075	1.0090	1.0976	1.0358	0.9742
Changjiang	0.9069	0.9692	0.9358	0.9878	0.9811
Ledong	0.9574	0.9880	0.9691	0.9651	1.0237
Lingshui	1.0311	1.0203	1.0107	1.0063	1.0138
Baoting	0.8940	1.0111	0.8842	0.9961	1.0151
Qiongzhong	0.9488	0.9602	0.9881	0.9814	0.9784
Mean	1.0003	1.0061	0.9942	1.0029	1.0031

### Effect of environmental factors on EPHS efficiency

The results of the LR test for the Tobit model (*p* < 0.001) showed that the random effects Tobit regression model was more suitable for the data than the mixed Tobit regression model. The maximum value of VIF was 2.80, indicating that there is no multicollinearity relationship among the environmental variables. As shown by the result of the Tobit regression, the bootstrapping bias-corrected technical efficiency of EPHS in Hainan Province was proportional to *per capita* GDP, with a regression coefficient of 0.0286, which was statistically significant (*p* < 0.05). The bootstrapping bias-corrected technical efficiency was inversely proportional to population density, the proportion of older people aged over 65, and the proportion of ethnic minority population, the regression coefficients of which were − 0.0003, −0.0316, and − 0.0041 respectively, which were statistically significant (*p* < 0.05) (Table 5).

**Table 5:** Tobit regression result.

	Regression coefficient	Std. Err	*p* value	VIF	Number of observations
*per capita* GDP	0.0286	0.0099565	0.004	2.07	216
Urbanization rate	−0.0012	0.0014468	0.425	2.80	216
Population density	−0.0003	0.0000918	<0.001	2.44	216
Proportion of people aged over 65	−0.0316	0.0067607	<0.001	1.75	216
Proportion of ethnic minority population	−0.0041	0.0007014	<0.001	1.93	216
Constant	1.1985	0.0927603	<0.001		
LR test	13.81		<0.001		

## Discussion

### The improvement of technical change could contribute to the increase of efficiency changes of EPHS and still needs to be pushed continuously

The annual changes in the efficiency of EPHS showed that efficiency had increased between 2011 and 2012 in Hainan Province. It was mainly attributed to the improvement in technical change caused by the release of the second edition of the “National Essential Public Health Service Standards” (NEPHSS) in 2011. Financed by the government, the “National Essential Public Health Service Program” was an important part of the healthcare reform launched in 2009, with the purpose of meeting basic public health needs through equalized services and improving the health level of the population (26). In 2009, the National Health Sector issued the first edition of the NEPHSS, which standardized and unified service behaviors and brought the public health services on track. Therefore, the pure technical efficiency of the 18 cities/counties in Hainan Province improved in 2010–2011, indicating that there was progress in the management level of EPHS. The second edition of the NBPHSS, released in 2011, explicitly proposed the requirement for electronic health records and regulated the collection, update, and storage of electronic health records. Electronic health records were a technological innovation for public health services, saving manpower costs, improving working efficiency (27), and contributes to the progress of technical change in EPHS in Hainan Province. Evidence from Spain also showed that health information technology could lead to more efficient and higher-quality health care (23).

However, the efficiency of EPHS in Hainan Province has been limited by technical changes after 2013, which is similar to the results of other studies on the efficiency of public health services in China (15, 19). The improvement of technical changes and EPHS requires targeted and refined policy support, including the construction of the health technician team and the utilization of technical innovation (28). Nevertheless, the health technicians working at the primary healthcare institutions in Hainan Province tended to provide poor services and technical skills, and the fiscal policy could not stimulate their enthusiasm for work. Moreover, no targeted policies for essential public health, especially those relevant to human resources policies, have been issued since 2013, which might be the reason why the technological changes in Hainan Province were less than 1 from 2013 to 2021 (except for 2018–2019).

### Allocation of resources mainly based on population size might lead to the uneven development of EPHS

There are two counties whose scale efficiency and pure technical efficiency were lower than the provincial average in Hainan Province, both of which were under the national poverty line.

In the aspect of scale efficiency, they did not reach the optimal scale of EPHS, which was determined by the essential public health demands in each county. The construction of health infrastructure was relatively weak in the two counties, resulting in a greater demand for health resources. However, a large component of essential public health financial investment in China is based on the number of residents in each region. Hainan Province follows this principle, with little consideration for the differences in economic development, demographic characteristics, and hygienic conditions among the 18 cities/counties. This allocation approach for health resources emphasizes equality instead of fairness. Equalization of EPHS was an important goal of the healthcare reform in 2009. Which means that all Chinese citizens have fair opportunities to receive EPHS, regardless of their gender, income level, and place of residence (29). However, imbalances in economic and social factors have hindered the fairness of EPHS, and the allocation of health resources based on population size apparently could not reduce the gap between wealthier and poorer regions. Regions with ample economic resources were more able to allocate sources to deliver EPHS, while economically underdeveloped regions did not get sufficient health resources. Owing to a lack of additional support to economically and medically disadvantaged regions, several counties had difficulty reaching the most appropriate scale of EPHS and meeting the public’s demand. Greek scholars also argued that resources should be redistributed, taking the necessities and needs of each health center under consideration (30). As to pure technical efficiency, the management levels of EPHS in the two counties were lower than the provincial average. The improvement of the management level requires a refined and efficient management system and professional management technicians. However, all these need resources in terms of labor, finance, and material. Thus, pure technical efficiency was also relatively low in the two counties due to an underdeveloped economic level and a lack of additional resources.

### Local demographic characteristics are also important factors in the efficiency of public health services

Environmental factors had an effect on the efficiency of EPHS in Hainan Province. Compared with other cities/counties, those with a higher proportion of older people and ethnic minorities had lower efficiency in EPHS. More older adult means a greater demand for health services. If there is insufficient investment in public health resources and health technicians are overloaded with work, the provision of EPHS will be affected, leading to a decrease in efficiency. In addition, staff from relevant departments in Hainan Province stated that the older people in Hainan Province did not utilize the EPHS as expected, resulting in poor utilization of health resources. A study carried out in Chile also found that regions with more people older than 65 years old would most probably be classified as inefficient regions (31).

There are six autonomous counties for ethnic minorities in Hainan Province, and ethnic minorities account for 17.8% (2022) of the population. Ethnic minority populations tend to live in areas with lower economic and hygienic conditions, and their access to public health care is limited by factors such as transportation, geographical location, education level, and cultural traditions. This poses a significant challenge to achieving equalization of EPHS in Hainan Province.

Based on the problems regarding the efficiency of EPHS in Hainan Province after the healthcare reform, this article presents the following suggestions. Firstly, the significance of technology and innovation in EPHS should be recognized. The improvement of technical changes requires both technical innovation and health technicians with advanced capabilities. Considering there no targeted policies for public health have been issued since 2013, Hainan Province is suggested to pay attention to the application of new technologies such as health information technology and the Internet in EPHS and establish a performance evaluation system that can motivate the health technicians. Secondly, Hainan Province should fully utilize the advantage of provincial management of counties to coordinate and plan the allocation of public health resources throughout the province. For cities/counties with poor economic development and low hygiene level, preferential policies in terms of health resources should be provided to ensure the provision of EPHS in the region. For cities/counties with developed economies and higher levels of sanitation, it is necessary to minimize the waste of resources while meeting the needs of EPHS. Thirdly, the EPHS of older people and ethnic minorities should be emphasized. It is recommended that Hainan Province strengthen the health education (32), improve the health literacy of the population, and take measures to address objective factors that limit access to EPHS.

## Conclusion

In this study, we used DEA to analyze the efficiency of EPHS in Hainan Province during 2010–2021. The main findings of this study were as follows: firstly, the technical change had led to an improvement in the efficiency of EPHS. However, technological innovation in EPHS still needs to be strengthened. Secondly, allocating health resources based on population size emphasized equality instead of fairness, which might lead to the uneven development of EPHS. Hainan Province is recommended to provide additional support for regions with poor economic development. Healthcare costs have been increasing worldwide as the prevalence of NCDs continues to rise (33). Lack of health resources and unbalanced development between regions are also faced by other developing countries. This study has provided references for these countries to properly mobilize and manage resources and provide affordable public health services for their citizens efficiently. Future study can focus on whether a policy or a measure actually improve the efficiency of EPHS, such as health information technology and additional support to economically disadvantaged regions.

### Limitations

This study still has some limitations. Firstly, the variables selected in this study were all based on the quantity of EPHS due to a lack of reliable methods to measure the quality of EPHS. Secondly, considering the DEA model’s requirements for the number of input and output variables, this study was unable to cover all the EPHS. Thirdly, the method of efficiency evaluation needs further improvement. Due to the lack of some data, this study used Tobit regression to analyze the impact of environmental factors on efficiency. However, the Tobit regression could not obtain the efficiency value after removing the impact of environmental factors.

## Data availability statement

The original contributions presented in the study are included in the article/Supplementary material, further inquiries can be directed to the corresponding authors.

## Author contributions

YT: Writing – original draft, Data curation. JP: Data curation, Writing – original draft. YL: Writing – review & editing. JH: Writing – review & editing.
