# Supplementary material for: Efficiency trends of essential public health services and possible influencing factors since the new round health reform in China: a case study from Hainan Province
Source: Front Public Health. 2023 Nov 6;11:1269473. doi: 10.3389/fpubh.2023.1269473 (PMC10657853; doi:10.3389/fpubh.2023.1269473)
Supplement: Supplementary file 1 [file Table_1.DOCX]

Appendix table 1 The annual means of environmental factors of 18 DMUs

|  | per capita GDP (USA$ 1,000) | urbanization rate (%) | population density (people/km^2^) | proportion of people aged over 65 (%) | proportion of ethnic minority population (%) |
| --- | --- | --- | --- | --- | --- |
| Haikou | 8.35 | 77.70 | 1080.30 | 7.53 | 2.06 |
| Sanya | 9.40 | 71.42 | 460.52 | 5.43 | 41.75 |
| Danzhou | 7.60 | 50.88 | 280.54 | 7.96 | 8.78 |
| Wuzhishan | 3.51 | 55.59 | 94.59 | 6.78 | 71.60 |
| Qionghai | 6.52 | 46.60 | 297.51 | 11.47 | 3.88 |
| Wenchang | 5.32 | 52.16 | 225.75 | 13.98 | 0.88 |
| Wanning | 4.90 | 45.10 | 289.61 | 9.72 | 17.66 |
| Dongfang | 5.19 | 45.84 | 190.44 | 7.56 | 20.72 |
| Ding’an | 4.14 | 41.63 | 240.37 | 11.02 | 2.18 |
| Tunchang | 3.70 | 43.59 | 212.87 | 9.55 | 11.73 |
| Chengmai | 7.84 | 49.79 | 236.42 | 10.35 | 1.21 |
| Lingao | 5.19 | 43.02 | 323.46 | 9.45 | 0.18 |
| Baisha | 3.87 | 34.08 | 79.91 | 7.77 | 65.21 |
| Changjiang | 6.77 | 53.23 | 142.51 | 8.80 | 40.86 |
| Ledong | 3.52 | 33.62 | 168.50 | 8.20 | 38.78 |
| Lingshui | 5.96 | 42.40 | 316.56 | 8.07 | 56.81 |
| Baoting | 4.14 | 35.95 | 133.07 | 8.28 | 68.72 |
| Qiongzhong | 3.60 | 34.59 | 66.74 | 8.38 | 62.13 |
| Mean | 5.53 | 47.62 | 268.87 | 8.91 | 28.62 |
